# MicroRNA profiling identifies miR-7-5p and miR-26b-5p as differentially expressed in hypertensive patients with left ventricular hypertrophy

**DOI:** 10.1590/1414-431X20176211

**Published:** 2017-10-19

**Authors:** C.M. Kaneto, J.S. Nascimento, M.C.R. Moreira, N.D. Ludovico, A.P. Santana, R.A.A. Silva, I. Silva-Jardim, J.L. Santos, S.M.B. Sousa, P.S.P. Lima

**Affiliations:** 1 Departamento de Ciências Biológicas, Universidade Estadual de Santa Cruz, Ilhéus, BA, Brasil; 2 Departmento de Ciências da Saúde, Universidade Estadual de Santa Cruz, Ilhéus, BA, Brasil; 3 Departmento de Ciências Naturais, Universidade Estadual do Sudoeste da Bahia, Vitória da Conquista, BA, Brasil; 4 Instituto Multidisciplinar em Saúde, Universidade Federal da Bahia, Vitória da Conquista, BA, Brasil

**Keywords:** Cardiovascular disease, Hypertension, Left ventricular hypertrophy, microRNAs

## Abstract

Recent evidence suggests that cell-derived circulating miRNAs may serve as biomarkers of cardiovascular diseases. However, a few studies have investigated the potential of circulating miRNAs as biomarkers for left ventricular hypertrophy (LVH). In this study, we aimed to characterize the miRNA profiles that could distinguish hypertensive patients with LHV, hypertensive patients without LVH and control subjects, and identify potential miRNAs as biomarkers of LVH. LVH was defined by left ventricular mass indexed to body surface area >125 g/m^2^ in men and >110 g/m^2^ in women and patients were classified as hypertensive when presenting a systolic blood pressure of 140 mmHg or more, or a diastolic blood pressure of 90 mmHg or more. We employed miRNA PCR array to screen serum miRNAs profiles of patients with LVH, essential hypertension and healthy subjects. We identified 75 differentially expressed miRNAs, including 49 upregulated miRNAs and 26 downregulated miRNAs between LVH and control patients. We chose 2 miRNAs with significant differences for further testing in 59 patients. RT-PCR analysis of serum samples confirmed that miR-7-5p and miR-26b-5p were upregulated in the serum of LVH hypertensive patients compared with healthy subjects. Our findings suggest that these miRNAs may play a role in the pathogenesis of hypertensive LVH and may represent novel biomarkers for this disease.

## Introduction

Cardiovascular disease has an important impact on morbimortality and in Brazil, the incidence of essential hypertension (EH), a common chronic disease, has diminished about 6% in the last three decades, but it still is approximately 30% (1). Essential hypertension may increase the risk of several cardiovascular diseases, including coronary heart disease, stroke, kidney failure and heart failure, all of which may have serious consequences for human health. In recent years, due to actual living conditions, progressive lengthening in life expectancy and stress, the prevalence of essential hypertension in young patients is increasing and there is urgent need for methods to prevent and early identify hypertension and its complications (2).

The heart responds to an increased pressure load or increased volume load with an increased mass of muscle, which is recognized as cardiac hypertrophy. The mass of the myocardium increases through an enlargement of the individual muscle fibers (2). This left ventricular hypertrophy (LVH) implies an increase in left ventricular mass through an increased wall thickness and/or an increased internal dimension of the ventricle. LVH was previously regarded as an adaptive remodeling. However, evidence shows excessively high LV mass is associated with LV systolic dysfunction being a predictor of cardiovascular events, suggesting LVH is an independent predictor of adverse prognosis (3,4). It was widely accepted that LVH was mainly driven by pressure overload, but there are evidence suggesting that the pathogenesis and regulation of LVH is complicated and not mediated only by the mechanical stress. Some additional hemodynamic factors play important roles in the development and maintenance of LV hypertrophy, a condition with variable background and primary intrinsic mechanisms, genetic factors and physiological adaption to physical training may be evolved (5).

To further elucidate the regulation of LVH and find new risk factors predicting LVH in patients are thus of great importance (4).

Molecular biomarkers could be of benefit to identify and stratify these patients and studies in the cardiovascular research field have demonstrated the vital roles of microRNAs for proper cardiovascular development and functional maintenance (3,5,6). The involvement of aberrant microRNA expression leading to development of cardiovascular diseases lends further support of the regulatory role of microRNAs in heart function (7–9). MicroRNAs (miRNAs) are single stranded, short length (21 to 23 nucleotides), non-coding RNA molecules that function as a negative posttranscriptional regulation mechanism and play a major regulatory role in various mechanisms in developmental biology, physiology and pathology of almost every organ including the cardiovascular system (10–13). MicroRNAs are also highly stable in blood, making circulating microRNAs attractive novel serum biomarkers (14,15) that can be detected with high sensitivity and specificity in plasma and serum.

LVH is extensively controlled and regulated by microRNAs (16,17) and to date, a few miRNAs have been identified to have a regulatory role in LVH, of which miR-1 and miR-133 are the most studied (9), but identifying novel miRNAs related to the pathogenesis of hypertension and LVH could eventually lead to the development of new diagnostic methods and treatment approaches for hypertension and LVH. In this context, the expression profile of a panel of microRNAs relevant to a pathway or disease state could be very informative and tools like PCR arrays could provide good sensitivity, reproducibility and specificity. In this work, we investigated the expression of 84 microRNAs in patients with hypertensive left ventricular hypertrophy (LVH), hypertensive patients without LVH (HAS) and healthy subjects (Control) in order to identify differentially expressed microRNAs. MiR-7-5p and MiR-26b-5p showed differential expression pattern in PCR array analysis and RT-PCR assays were performed to validate their expression levels. MiR-26b was also previously identified as a modulator of physiological cardiac hypertrophy (18) and we hypothesized that circulating levels of some microRNAs, like MiR-7-5p and MiR-26b-5p, could be used as plasma biomarkers that predict LVH in patients with essential hypertension.

## Material and Methods

### Patients

In the matched case-control study, we recruited 3 hypertensive patients with left ventricular hypertrophy (LVH), 4 hypertensive patients without LVH (HAS) and 4 healthy subjects (Control) to perform miScript miRNA PCR array analysis. For large sample validation, 8 hypertensive patients with LVH aged 55-60 years (average 57.6±2.51 years), 28 hypertensive patients without LVH aged 37–65 years (average 52.8±9.61 years) and 23 healthy subjects aged 41–55 years (average 46.8±4.7 years) were recruited and included the samples used in PCR array analysis. In order to minimize age influence on microRNA expression, we have included patients with a high age range in all three groups of the study. All patients with LVH had previous diagnosis of hypertension, all hypertensive patients were under treatment and had blood pressure control in the last 6 months. The medication used for hypertension and LVH treatment are shown in Table 1. Blood samples of all subjects were collected. All echocardiographic studies were performed using a commercial ultrasound machine (HD 11, Philips, USA). LV end-diastolic and -systolic diameters were measured and indexed to the body surface area, according to American Society of Echocardiography guidelines. As an additional simple estimate of LVH, the maximal wall thickness (MWT) measured at any level in the LV wall was also considered. LVH was defined by left ventricular mass indexed to body surface area >125 g/m^2^ in men and >110 g/m^2^ in women. Patients were classified as hypertensive according to WHO (World Health Organization) criteria (2) when presenting a systolic blood pressure (SBP) of 140 mmHg or more, or a diastolic blood pressure (DBP) of 90 mmHg or more. LVH, hypertensive and control subjects had no other concomitant diseases, including another form of hypertension in addition to essential hypertension, body mass index greater than 35 kg/m^2^, cancer, heart valve disease, acute coronary artery disease or acute myocardial infarction, Chagas disease, bundle branch block and ventricular pre-excitation syndromes. The institutional Ethics and Clinical Research Committee of the Universidade Estadual de Santa Cruz, Ilhéus, BA, Brazil, approved the study and all patients gave written informed consent.

**Table 1.** Clinical characteristics of the patients.

Characteristics	Control subjects	Hypertensive subjects	Left ventricular hypertrophy hypertensive subjects	P value
n	23	28	8	
Age (years)	>46.8±4.7	>52.8±9.6	>57.6±2.5	0.12
SBP (mmHg)	>106.6±5.7	>126.6±5.7	>123.3±5.8	0.0005*
DBP (mmHg)	>70±10	>83.3±5.7	>86.3±5.7	0.07
LV mass index (g/m^2^)	>55.8±10.2	>61.8±9.1	>158.5±67.9	<0.0001*
LV wall thickness (mm)	>9.33±0.57	>8.88±0.83	>13.0±1.88	0.0002*
LV dimension (mm)	>31.3±4.6	>32.2±2.8	>38.2±3.11	0.01*
Ejection fraction (%)	>74.6±5.5	>71.2±6.9	>69.3±5.6	0.5
Glucose (mg/dL)	>79.3±13	>88.2±11.4	>88.6±5.2	0.14
Creatinine (mg/dL)	>0.75±0.14	>0.87±0.15	>0.80±0.2	0.23
Uric acid (mg/dL)	>4.26±1.8	>4.27±0.5	>3.9±0.7	0.83
Current smoker (N)	2	2	3	
Medication				
ACEI/ARBs (%)		75	100	
B-blockers (%)		25	20	
Diuretic (%)		25	25	

SBP: systolic blood pressure; DBP: diastolic blood pressure; LV: left ventricular; ACEI: angiotensin converting enzyme inhibitor; ARB: angiotensin receptor blocker.

* P<0.05.

### Blood samples

Blood was collected from each subject into EDTA anticoagulant tubes that were spun in a Hettich Universal 320R centrifuge at 1300 *g* for 15 min at room temperature, with no brake applied, to obtain plasma. The upper (plasma) phase was collected to above 5 mm of the buffy coat for each sample. Platelet-poor plasma was obtained by distributing the pooled sample into clean 15 mL conical centrifuge tubes and re-spinning at 3220 *g* for 10 min at room temperature with a brake setting of 9. The supernatant was transferred to 1.5 mL microcentrifuge tubes and stored at –80°C until use.

### RNA isolation and complementary DNA (cDNA) synthesis

Total RNA was isolated using TRIzol LS (Invitrogen, USA) and miRNeasy Mini Kit (Qiagen, USA) according to the manufacturer’s instructions. For each RNA extraction, 250 μL once-thawed plasma added to 750 μL TRIzol LS (Invitrogen, USA). The solution was vortexed at high speed for 15 s and incubated at room temperature for 5 min. Synthetic sequences (1 μL) from the RNA spike-in kit (Qiagen) were added, followed by 200 μL of chloroform. Each tube was vortexed vigorously for 30 s and allowed to sit at room temperature for 5 min. Phase separation was achieved by centrifuging the sample at 12,000 *g* for 20 min at 4°C. After centrifugation, 400 μL of the aqueous phase was carefully transferred to a new tube for spin column purification. RNA was eluted in nuclease-free water by passing a few times through a pipette tip. RNA quality and quantity were measured by using Nanodrop spectrophotometer (ND-1000, Nanodrop Technologies) and RNA integrity was determined by gel electrophoresis. Total RNA that contains miRNA (600 ng) was used for cDNA synthesis using miScript II RT Kit (Qiagen) using miScript Reverse Transcriptase Mix, 10× miScript Nucleics Mix, and 5× miScript HiSpec Buffer. The mixture was incubated for 60 min at 37°C and for 5 min at 95°C to inactivate miScript Reverse transcriptase mix and placed on ice. This cDNA was diluted in RNase free water (20 μL of cDNA obtained above mixed with 180 μL of water).

### miRNA profiling

miRNA profiling was performed using Human miScript miRNA PCR array (96 well format, MIHS-113Z, Qiagen). The array profiles the expression of the 84 miRNAs known to exhibit altered expression during cardiovascular disease development. A set of controls are included on each plate which enabled data analysis using the 2^-ΔΔCT^ method of relative quantification, assessment of reverse transcription performance and assessment of PCR performance. The miScript miRNA PCR array enables SYBR Green-based real-time PCR analysis using ABI7500 fast real-time PCR system (Applied Biosystems, USA) as follows: 95°C for 15 min; 40 cycles of 94°C for 15 s; 55°C for 30 s, and 70°C for 30 s. The Web-based miScript miRNA PCR array data analysis tool (available at http://pcrdataanalysis.sabiosciences.com/mirna/arrayanalysis.php) was used to analyze the real-time PCR data. This tool calculates the fold change using ΔΔCT method of relative quantification.

### Validation of miR-7-5p and miR-26b-5p expression by real-time PCR

To validate the levels of miRNA selected from miRNA profile in wide range of samples, we used Taqman MicroRNA Assays (Applied Biosystems). qRT-PCR amplification mixtures contained 20 ηg template cDNA, Taqman master mix (10 μL; Applied Biosystems) and probes for MiR-7-5p (Cat. #4427975) and MiR-26b-5p (Cat. #4427975) in a final volume of 20 μL. All reactions were run in duplicate on an ABI7500 fast real-time PCR system (Applied Biosystems) under standard thermal cycling conditions. The mean cycle threshold (*Ct*) values from duplicate measurements were used to calculate expression of the target gene, with normalization to an internal control miR-342-3p (Cat. #4440886) using the 2^-ΔCT^ formula and presented as fold change (19–21). Experiments with coefficients of variation greater than 5% were excluded. A no-template control (NTC) and no reverse transcription controls (No-RT) were also included.

### Statistical analysis

All data were analyzed using the Prism 5.01 computer software (GraphPad, USA). Parametric data were analyzed using one-way ANOVA with Tukey’s *post ho*c. Statistical differences were considered to be significant at P*<*0.05. The relationship between the two circulating miRNAs and LV mass index were evaluated using Pearson correlation analysis.

## Results

### Patients

To characterize the miRNA profile of left ventricular hypertrophy, we recruited 3 hypertensive patients with left ventricular hypertrophy (LVH), 4 hypertensive patients without LVH (HAS) and 4 normal subjects as the control. The characteristics of the participants are shown in Table 1. There were no significant differences in age or blood pressure between the three groups. The LV mass index was dramatically higher in LVH hypertensive patients than controls.

### Identification of miRNAs expression patterns of patients with LVH

**Figure 1.**
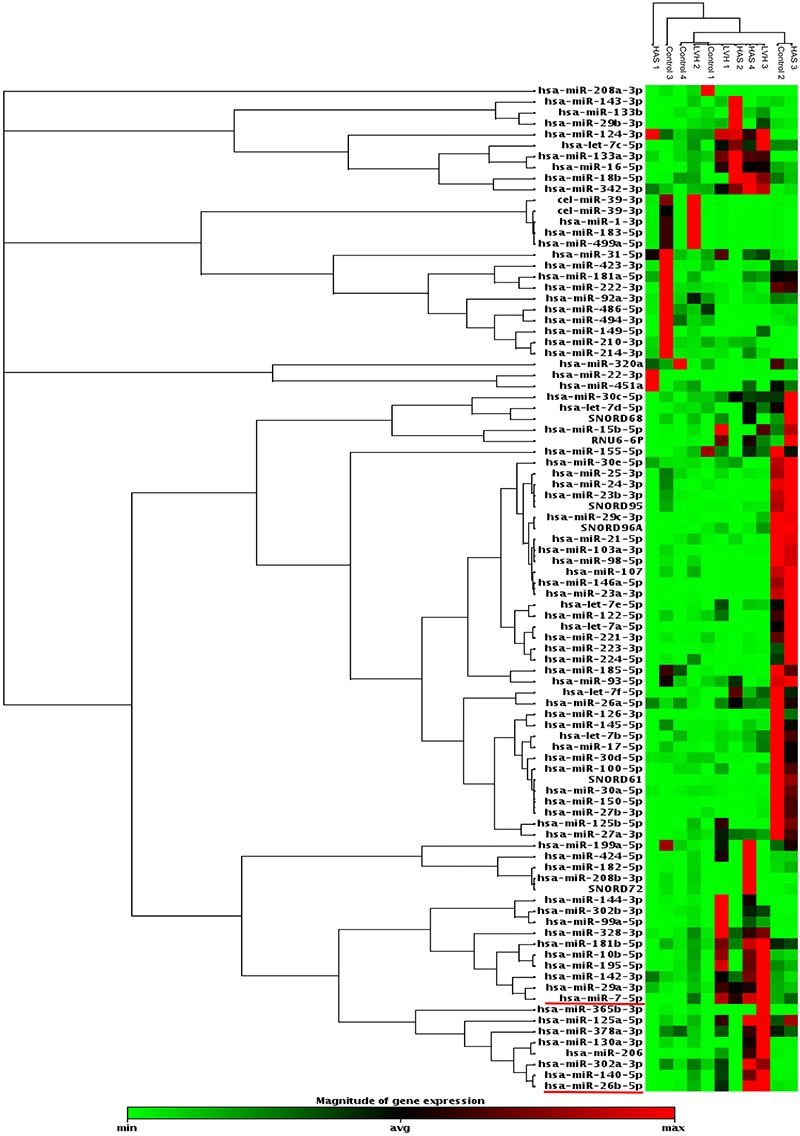
Heat map illustrating the expression patterns of upregulated and downregulated miRNAs in hypertensive patients with left ventricular hypertrophy (LVH). Upregulated miRNAs are indicated by red while downregulated miRNAs are indicated by green. The two candidate miRNA markers miR-7-5p and miR-26b-5p are underlined in red.

**Figure 2.**
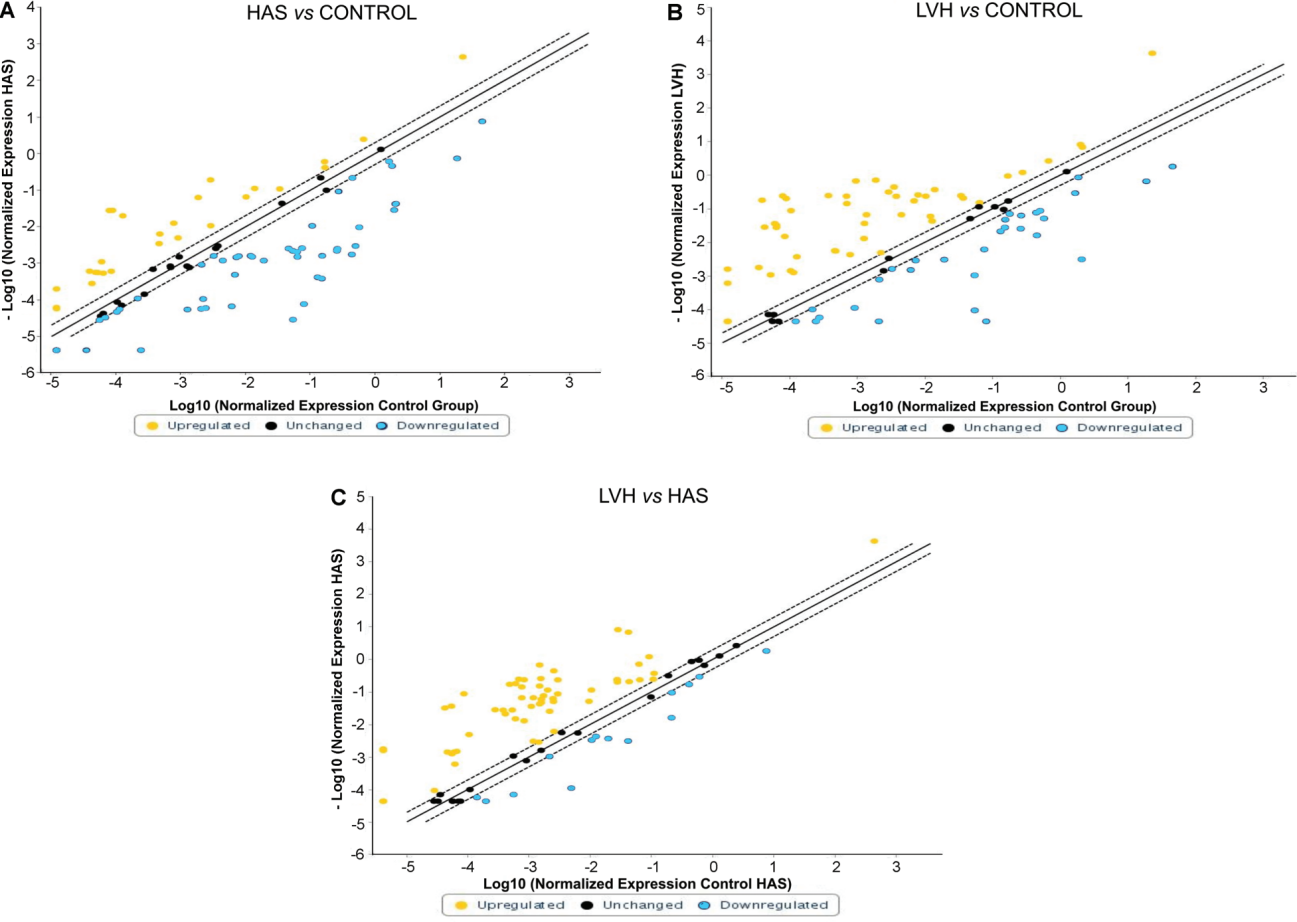
miRNAs differentially expressed in *A*, hypertensive patients without left ventricular hypertrophy (HAS) and control subjects; *B*, hypertensive patients with left ventricular hypertrophy (LVH) and control subjects and *C*, hypertensive patients with left ventricular hypertrophy (LVH) and hypertensive patients without left ventricular hypertrophy (HAS). The scatter plots illustrated miRNAs differentially expressed: dots in black indicate the miRNAs that did not reach significant changes of expression; dots in yellow indicate the miRNAs that had significant upregulation of expression; and dots in blue indicate the miRNAs that had significant downregulation of expression.

We performed microarray analysis to identify miRNAs expression patterns of hypertensive patients with left ventricular hypertrophy. We made a heat map to visualize the results of the two-way hierarchical clustering of miRNAs (Figure 1). The color scale shown at the bottom illustrated the relative expression level of a miRNA: red represented a high relative expression level while green represented a low relative expression level (22). Furthermore, a Scatter plot was made to illustrate miRNAs differentially expressed between healthy, hypertensive and left ventricular hypertrophy subjects (Figure 2). We identified 75 miRNAs differentially expressed between left ventricular hypertrophy and control, including 49 miRNAs upregulated and 26 miRNAs downregulated and 69 miRNAs differentially expressed between HAS and control, including 24 miRNAs upregulated and 45 miRNAs downregulated (Supplementary Table S1). We also identified 70 miRNAs differentially expressed between hypertensive left ventricular hypertrophy and HAS, including 56 miRNAs upregulated and 14 miRNAs downregulated.

### Confirmation of different miRNAs in serum of hypertensive patients with LVH

**Figure 3.**
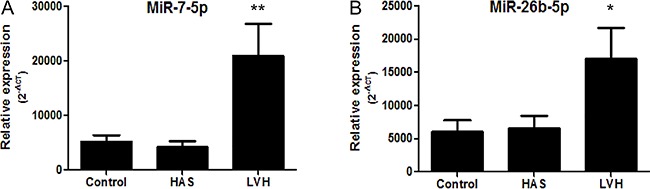
Circulating levels of miR-7-5p and miR-26b-5p in hypertensive patients with left ventricular hypertrophy (LVH) and hypertensive patients without left ventricular hypertrophy (HAS) patients and controls subjects, evaluated by Taqman real-time PCR (arbitrary units). *A*, Circulating level of miR-7-5-p, and *B*, circulating level of miR-26b-5p in healthy controls, hypertensive patients and patients with left ventricular hypertrophy. Data are reported as means±SE of 8 to 28 subjects/group. *P<0.05, **P<0.0001, compared to control subjects.

**Figure 4.**
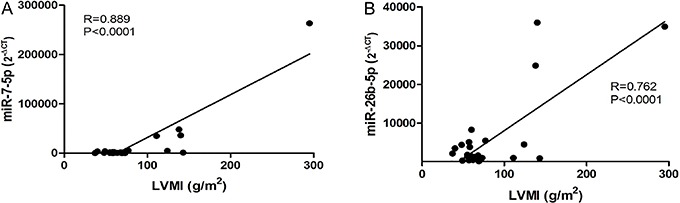
Left ventricle mass index (LVMI) and circulating level of miR-7-5p and MiR-26b-5p show strong correlation. *A*, Correlation between LVMI and MiR-7-5p. *B*, Correlation between LVMI and MiR-26b-5p.

To confirm our results, we chose two of the most differentially expressed upregulated miRNAs, miR-7-5p and miR-26b-5p, which showed differences of more than 300-fold between LVH, HAS and control groups. We enrolled 28 hypertensive patients without LVH, 8 hypertensive patients with LVH, and 23 healthy subjects. As shown in Figure 3, RT-PCR analysis showed that circulating serum levels of miR-7-5p were profoundly elevated in patients with LVH compared to healthy subjects and HAS patients (P<0.0001). Circulating levels of miR-26b-5p were also significantly increased in hypertensive patients with LVH compared to healthy subjects and HAS patients (P<0.05). The relationship between the two circulating miRNAs and LV mass index were evaluated and showed that LV mass index was closely correlated with miRNAs expression. As for the two circulating miRNAs, miR-7-5p showed the strongest correlation with LV mass index (r=0.889, P<0.0001). MiR-26b-5p also had a correlation with LV mass index (r=0.762, P<0.0001; Figure 4). These data confirmed our results of PCR array analysis.

## Discussion

LVH is associated with cardiovascular morbidity and mortality, as well as all-cause mortality (23). LVH is also independently associated with the risk of ischemic stroke and other cardiovascular complications. Although hypertension is known to be a major cause of LVH, the degree of LVH is not in parallel with the level of BP (blood pressure), the duration of hypertension or reversal of hypertensive LVH by pharmacological treatment. This implies that other factors may be involved in LVH in addition to BP (24). Despite extensive and continuous efforts to understand the pathogenesis of essential hypertension and LVH, the underlying cellular and molecular mechanisms remain largely elusive. Thus, it is essential to identify these factors, selecting the population at risk, as well as to establish the early diagnosis of LVH. This information could help the clinician not only to make decisions about the timing of pharmacological therapy for asymptomatic patients but also in the reinforcement of adjuvant measures (weight loss, arterial pressure control, etc.) aimed at improving the outcome in all HAS and LVH hypertensive patients.

Emerging evidence suggests that miRNAs are pivotal regulators of various processes including cell proliferation, differentiation, apoptosis, survival, motility, and morphogenesis (25). miRNAs are a class of short, noncoding, single stranded RNA molecules, approximately 22 nucleotides in length that negatively regulate gene expression either through inhibition of mRNA translation or by promoting mRNA degradation. Recently, specific miRNA expression profiles have been reported as a prognostic factor or a predictive factor for disease progression. Specifically, serum miRNAs may be used as biomarkers in diagnosis (26). In pathological process, miRNAs are linked to a variety of cardiovascular disease, including myocardial hypertrophy, myocardial fibrosis, heart failure and arrhythmias. Fang et al. (7) demonstrated that 14 circulating miRNAs are associated with diffuse myocardial fibrosis and suggested that circulating miRNAs could be a favorable alternative for assessing diffuse myocardial fibrosis because of its convenience, with no contraindications. Roncarati et al. (8) suggest that cardiac remodeling associated with hypertrophic cardiomyopathy (HCM) determines a significant release of miRNAs into the bloodstream: the circulating levels of both cardiac and non-cardiac-specific miRNAs are significantly increased in the plasma of HCM patients. However, correlation with left ventricular hypertrophy parameters holds true for only a few miRNAs, whereas only miR-29a is significantly associated with both hypertrophy and fibrosis, identifying it as a potential biomarker for myocardial remodeling assessment in HCM (8). Expression levels of miR-208, associated with cardiac hypertrophy by negative regulation of SOX6, were also found significantly increased in the peripheral blood of patients with left ventricular hypertrophy compared with those of healthy individuals, suggesting that it may be useful as a novel therapeutic and diagnostic marker for cardiac hypertrophy (8).

Therefore, we hypothesized that in LVH serum miRNA levels could be changed and those miRNAs could be used as the biomarkers. In this study, we performed PCR array analysis to identify 75 miRNAs differentially expressed in the serum of hypertensive patients with LVH, including 49 miRNAs upregulated and 26 miRNAs downregulated. After a rigorous selection, we screened 2 differentially expressed miRNAs, miR-7-5p and miR-26b-5p. Further large sample validation in 59 patients demonstrated a distinct serum microRNA expression pattern in hypertensive patients with LVH: Both microRNAs were upregulated compared with hypertensive patients without LVH and control subjects.

MiR-7-5p is found to be downregulated in cancer cells (27) and have been reported to be “tumor suppressor”. Interestingly, one of the validated targets of miR-7-5p is EGFR (epidermal growth factor receptor) mRNA (28–30) which is a major inducer of cell proliferation. MiR-7-5p is also suggested to inhibit vascular endothelial cell proliferation by targeting RAF1, inhibiting angiogenesis.

Changes in miR-26b-5p were also observed in some types of cancer (31–33) suppressing cell migration, invasion, metastasis, angiogenesis and apoptosis, suggesting a promising application for miR-26b-5p in anti-cancer therapy. Wu et al. (34) validated and confirmed the dysregulation of miR-26b-5p in pulmonary arterial hypertension (PAH), suggesting that it could serve as a potential biomarker for PAH and miR-26b-5p expression was also found to be altered in patients with acute heart failure (35), but there are no studies regarding these microRNAs expression and hypertensive LVH, so their function in cardiovascular diseases need further studies.

In conclusion, the current study suggested circulating levels of miR-7-5p and miR-26b-5p were elevated in LVH hypertensive patients and further prospective investigations are needed to elucidate whether these circulating microRNAs affect clinical outcome. Evaluation of the screened up- and downregulated microRNAs according to their target mRNAs and biological significance will give some clues for their functional role in hypertensive LVH. Our findings suggested that these miRNAs may have a role in the pathogenesis of LVH associated with HAS and may represent novel biomarkers for the diagnosis and prognosis of LVH. These findings were of great importance because circulating microRNAs can be easily measured and thus potentially have wide clinical applications. Therefore, prevention of events, including the accurate identification of risk factors, remains a challenge for public health.

## Supplementary material

Click here to view [pdf].
